# Interaction between *GSTM1*/*GSTT1* Polymorphism and Blood Mercury on Birth Weight

**DOI:** 10.1289/ehp.0900731

**Published:** 2009-10-23

**Authors:** Bo-Eun Lee, Yun-Chul Hong, Hyesook Park, Mina Ha, Bon Sang Koo, Namsoo Chang, Young-Man Roh, Boong-Nyun Kim, Young-Ju Kim, Byung-Mi Kim, Seong-Joon Jo, Eun-Hee Ha

**Affiliations:** 1 Department of Preventive Medicine, School of Medicine, Ewha Woman’s University, Seoul, Korea; 2 Department of Preventive Medicine, Seoul National University College of Medicine, Seoul, Korea; 3 Department of Preventive Medicine, Dankook University College of Medicine, Cheonan, Korea; 4 Department of Obstetrics and Gynecology, Ulsan University Hospital, University of Ulsan College of Medicine, Ulsan, Korea; 5 Department of Nutritional Science and Food Management, Ewha Woman’s University, Seoul, Korea; 6 Institute of Environmental and Industrial Medicine, Hanyang University, Seoul, Korea; 7 Division of Child and Adolescent Psychiatry, Department of Psychiatry and Institute of Human Behavioral Medicine, Seoul National University College of Medicine, Seoul, Korea; 8 Department of Obstetrics Medicine, School of Medicine, Ewha Woman’s University; 9 Ministry of Environment, Division of Environmental Health Policy, Gwacheon, Korea

**Keywords:** birth weight, GSTM1, GSTT1, mercury, polymorphism, pregnancy

## Abstract

**Background:**

Mercury (Hg) is toxic to both the reproductive and nervous systems. In addition, glutathione *S*-transferases (GSTs), which conjugate glutathione to a variety of electrophilic compounds, are involved in the detoxification of Hg.

**Objective:**

In this study we examined the association between prenatal exposure to Hg and birth weight as well as the influence of GST polymorphisms.

**Methods:**

The total Hg concentration in maternal and cord blood was measured from 417 Korean women and newborns in the Mothers and Children’s Environmental Health study from 2006 to 2008. Information on birth weight was collected from the patients’ medical records. The genotyping of glutathione *S*-transferase M1 (*GSTM1*) and glutathione *S*-transferase T1 (*GSTT1*) polymorphisms was carried out using polymerase chain reaction. Regression analysis was performed to determine the association between the blood Hg concentration and birth weight in mothers with *GSTM1* and *GSTT1* polymorphisms.

**Results:**

The geometric mean levels of Hg in the maternal blood during late pregnancy and in cord blood were 3.30 μg/L and 5.53 μg/L, respectively. For mothers with the *GSTT1* null genotype, elevated Hg levels in maternal blood during late pregnancy were associated with an increased risk of lower birth weight. For mothers with both *GSTM1* and *GSTT1* null genotype, both maternal and cord blood Hg levels were associated with lower birth weight.

**Conclusions:**

This study suggests that the interactions of Hg with *GSTM1* and *GSTT1* polymorphisms play a role in reducing birth weight.

Mercury (Hg) is ubiquitous in the global environment with both natural and anthropogenic origins (Gundacker et al. 2007; Oken and Bellingerb 2008). There has been increasing concern regarding the detrimental effects of Hg on human health (Counter and Buchanan 2004). Hg has toxic effects on a wide variety of systems, particularly the central nervous system, kidneys, and skin (Brodsky et al. 1985). Moreover, exposure to methylmercury (MeHg) can have neurotoxic effects through oxidative stress (Castoldi et al. 2001). In particular, pregnant women, fetuses, and infants are quite susceptible to the toxic effects of Hg (Jedrychowski et al. 2006; Trasande et al. 2005). Previous studies have reported that intrauterine exposure to total Hg is associated with neurobehavioral dysfunction in children (Jedrychowski et al. 2006; Lederman et al. 2008).

In addition, the reproductive toxicity of MeHg and Hg vapor has been confirmed in many animal studies (Beyrouty and Chan 2006; Fredriksson et al. 1993; Morgan et al. 2002). In humans, several studies have reported a relationship between occupational Hg exposure and adverse reproductive outcomes, such as spontaneous abortion, growth retardation, congenital malformation, and fetal death (Seidler et al. 1999; Sikorski et al. 1987). However, studies examining Hg exposure in relation to birth weight have shown conflicting results. Some studies have reported an inverse association between the birth weight and the MeHg concentration in maternal and cord blood (Foldspang and Hansen 1990) and total Hg levels in the newborn and maternal hair (Sikorski et al. 1986; Xue et al. 2007), whereas others have shown no such association (Grandjean et al. 2001; Lederman et al. 2008; Lucas et al. 2004; Marques et al. 2008). This variability may be mediated partly by genetic variations.

It has been suggested that glutathione (GSH) plays an important role in Hg metabolism (Custodio et al. 2005). Both inorganic Hg and MeHg bind to GSH, possibly through a process catalyzed by glutathione *S*-transferase (GST) (Engström et al. 2008; Gundacker et al. 2007), and stable GSH–metal conjugates are eliminated in the feces and urine (Ballatori 2002; Ballatori and Clarkson 1985). GSTs are phase II xenobiotic metabolizing enzymes that catalyze the conjugation of a variety of electrophilic compounds with GSH (Hayes and Strange 2000). Glutathione *S*-transferase M1 (*GSTM1*) and glutathione *S*-transferase T1 (*GSTT1*) genes are polymorphic in the human population (Schneider et al. 2004; Wormhoudt et al. 1999). *GSTM1* and *GSTT1* null genes are the result of two homozygous deletions that result in a loss of functional activity (Jain et al. 2006). However, there is inconsistency in the relationship between *GSTT1* and *GSTM1* polymorphisms and Hg level. Gundacker et al. (2007) reported that the double-deleted homozygous genotypes for *GSTT1* and *GSTM1* were associated with a higher hair Hg levels, whereas the *GSTM1*/*GSTT1* genotype was not related to the retention of MeHg, as measured by the erythrocyte total Hg or inorganic Hg in the whole blood, plasma, and urine (Custodio et al. 2004, 2005).

On the other hand, Hg exposure causes oxidative stress through the formation of free radicals or an alteration of the antioxidant capacity of cells (Hussain et al 1997; Lund et al. 1993), and Hg-induced oxidative stress might interfere with normal intrauterine growth (Kim et al. 2005). However, GSH may scavenge the reactive oxygen species generated by Hg exposure (Pinheiro et al. 2008), and the *GSTM1* (Park et al. 2008) and *GSTT1* (Dusinská et al. 2001) null genotypes have been associated with increased oxidative stress.

To date, the relationships among Hg exposure, birth weight, and GST polymorphisms have not yet been examined. Therefore, this study examined whether a GST polymorphism can modify the association between intrauterine Hg exposure and birth weight.

## Materials and Methods

### Study population and data collection

This study is based on the Mothers and Children’s Environmental Health (MOCEH) study, which is a multicenter prospective cohort study. The MOCEH study has been carried out since 2006 to determine the effects of maternal environmental exposure on fetal and postnatal growth or development. All pregnant women living in the targeted study site (i.e., Seoul, Cheonan, and Ulsan) who are in the first trimester of pregnancy at the time of screening are eligible. The study protocol was approved by the institutional review boards at Ewha Woman’s University (Seoul), Dankook University Hospital (Cheonan), and Ulsan University Hospital (Ulsan, South Korea), and written informed consent was obtained from each woman.

At the time of this study, 957 pregnant women were enrolled in the MOCEH study, and the pregnancy outcomes of 629 women were followed up. The study subjects were restricted to those for whom maternal and cord blood Hg levels and *GSTM1*/*GSTT1* genotype were assessed. Overall, 445 participants were eligible for this study. Of these 445 women, the following subjects were excluded: 14 with multiple births, 1 with stillbirth, 1 whose infant had a congenital anomaly, and 12 who had preeclampsia or gestational diabetes mellitus. Finally, 417 pregnant women were enrolled in the analysis.

Data collection consisted of an interviewed questionnaire, nutritional survey, and biological samples (blood and urine). Information on sociodemographic characteristics, prior medical history, psychosocial status, health behavior, and environmental exposure was collected from an interview with trained nurses. Gestational age was estimated based on the onset of the last menstrual period; if the last menstrual period was unreliable or if there was a significant discordance between the ultrasonographic and last menstrual period dating (> 10 days), the first ultrasonographic estimation of the gestational age was used. Trained nurses in the delivery room routinely measured birth weight using a digital scale at birth and recorded it on the patient’s medical charts. Information on the birth outcome, such as birth weight, gestational age, parity, and infant sex, was collected from the medical records at delivery. A dietary assessment based on 24-hr recall of dietary intake on the day before the blood test was administered within 20 gestational weeks. The food and nutrient intake, including the amount of fish consumption, was assessed using a computerized nutrient-intake assessment software program (CAN-Pro 3.0; Korean Nutrition Society, Seoul, Korea).

### Hg analysis

The maternal blood samples were obtained during early pregnancy (12–20 gestational weeks) and late pregnancy (28–42 gestational weeks). The cord blood samples were collected at birth. The blood samples were stored at −70°C until analysis. Hg analysis was performed by flow injection cold-vapor atomic absorption spectrometry (AAS) (DMA-80; Milestone, Bergamo, Italy). The sample was initially dried in an oxygen stream passed through a quartz tube located inside a controlled heating coil. The combustion gases were further decomposed on a catalytic column at 750°C. Hg vapor was collected on a gold amalgamation trap and then desorbed for quantification. The Hg content was determined by AAS. The laboratory analyses were carried out using standardized quality-control procedures. An internal control was used for each series of analyses. The precision and accuracy of the Hg level measurement were verified by periodically participating in an external quality control program (interlaboratory calibration exercises). The limit of detection (LOD) was 0.158 μg/L, and no sample had an Hg level below the LOD.

### Genotyping of GSTM1 and GSTT1

The genomic DNA was extracted from the whole blood using a QIAamp DNA blood kit (Qiagen, Valencia, CA, USA). *GSTM1* and *GSTT1* polymorphisms were genotyped using a polymerase chain reaction (PCR) approach. As a positive control, coamplification of the 268-bp fragment of the β-globin gene was performed at the same time as the analysis of the *GSTM1* and *GSTT1* polymorphisms. The PCR mixture (20 μL) for *GSTM1* and *GSTT1* genotyping contained 10 mM Tris-HCl (pH 9.0), 40 mM KCl, 1.5 mM MgCl_2_, 0.25 mM of each dNTP, 1 unit *Taq* polymerase (Bioneer, Seoul, Korea), 20 pmol of the forward and reverse primers, and 50–100 ng of the genomic DNA as a template. The following primer sets for the *GSTM1* and *GSTT1* genes were used for the amplification reaction: 5′-GAACTCCCTGAAAAGCTAAAGC-3′ (forward) and 5′-GTTGGGCTCAAATATACGGTGG-3′ (reverse) for *GSTM1*, and 5′-TCACCGGATCATGGCCAGCA-3′ (forward) and 5′-TTCCTTACTGGTCCTCACATCTC-3′ (reverse) for *GSTT1*. The amplifications were performed under the following conditions: initial denaturation at 94°C for 5 min; 35 cycles of denaturation at 94°C for 1 min, annealing at 65°C for 1 min, and extension at 72°C for 1 min; and a final extension at 72°C for 7 min. PCR amplification of the reaction mixtures was carried out using a PTC-200 thermal cycler (MJ Research, Watertown, MA, USA).

To evaluate the PCR-amplified fragments, electrophoresis on 3% 3:1 NuSieve/agarose gel was used (Cambrex Bio Science, Rockland, ME, USA). Genotyping of the *GSTM1* and *GSTT1* genes was performed based on the presence of a 215-bp product and 459-bp product, respectively. The null genotype was defined as a homozygous deletion of the gene. To confirm the analyses, 10% of the samples were selected randomly and genotyped again, with identical results.

### Statistical analysis

Descriptive analyses were given for characteristics of the study subjects, and birth weight according to maternal and infant characteristics was evaluated using *t*-test or analysis of variance. After descriptive analyses, Hg concentrations were transformed logarithmically because of their skewed distribution. The geometric means (GMs) and percentiles for Hg concentrations were calculated. The relationship between Hg concentration and risk factors were examined using a Wilcoxon rank-sum test or Kruskal–Wallis rank sum test.

We used regression analysis to examine the effects of Hg levels in the maternal and cord blood on the birth weight in mothers with the *GSTM1* or *GSTT1* genotype. The covariates in the multiple regression models were chosen as follows: First, the risk factors associated with Hg exposure or birth weight were identified from the literature. Second, the variable was considered to be a potential confounder if the variables were related to birth weight or Hg level at *p* < 0.20 in univariate analyses. Covariates included the indicator variables for infant sex, maternal age (< 30, 30 to < 35, ≥ 35 years), prepregnancy body mass index (BMI; < 18.5, 18.5 to < 23, ≥ 23 kg/m^2^), maternal educational level (< 12, ≥ 12 years), parity (0, 1, ≥ 2), and continuous variables for gestational age (weeks) and weight gain (kilograms). In addition, information on amalgam fillings was collected, but there was no correlation between the presence of amalgam fillings and blood Hg levels. Therefore, amalgam fillings were not included in the analyses. Some participants had missing data for a particular variable and were excluded from multivariate analysis. To examine interactive effect of the genotypes and Hg on birth weight, we included an interaction term between Hg level and genotypes in the multivariate analysis. After identifying the interaction effect, stratified analysis was performed by genotypes of *GSTM1*/*GSTT1*, and combined effects of *GSTM1* and *GSTT1* genotype were also assessed using regression analysis.

In the generalized linear model, analysis of covariance was carried out for birth weight, and the least-square mean of birth weight according to *GSTM1* and *GSTT1* polymorphism and Hg level was estimated. The average Hg level during pregnancy was divided into three categories according to a tertile distribution, and *GSTM1*/*GSTT1* genotype was classified as both present, either null, and double null. All statistical analyses were carried out using SAS statistical software (version 8.2; SAS Institute Inc., Cary, NC, USA).

## Results

All participants were of Korean ethnicity. The mean maternal age was 30.1 years, and 66.3% of the participants had completed > 12 years of education. The prevalence of the null genotype for *GSTM1* and *GSTT1* was 59.5% and 53.2%, respectively. The mean ± SD birth weight for newborns was 3,282 ± 433 g. Approximately 97% of the infants were born at term. The birth weight increased with increasing BMI at prepregnancy and gestational age (Table 1).

Table 2 shows the distribution of Hg concentrations. The GM of the maternal blood Hg concentration was 3.67 μg/L during early pregnancy and 3.30 μg/L during late pregnancy. The levels of cord blood Hg were higher than the concentration of maternal blood Hg.

**Figure 1:**
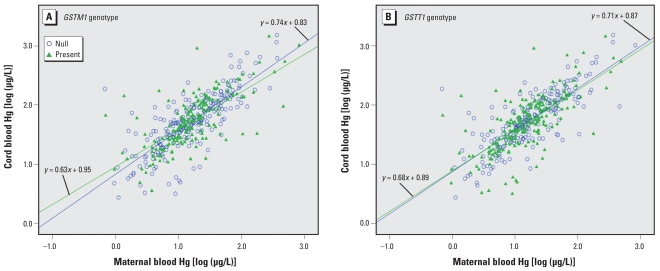
Relationship between cord blood and maternal blood Hg by *GSTM1* (*A*) and *GSTT1* (*B*) genotype.

Hg levels in the cord blood correlated with that in the maternal blood during late pregnancy (Pearson *r* = 0.72, *p* < 0.0001). When we analyzed the relationship between maternal and cord blood according to *GSTM1* and *GSTT1* genotype (Figure 1), we found that the regression lines by *GSTM1* genotype were somewhat different but did not reach statistical significance (*p* = 0.59). For *GSTT1* genotype, we observed no significant difference (*p* = 0.91).

As shown in Table 3, maternal blood Hg in late pregnancy and Hg in cord blood were significantly higher in women with a higher educational level. Hg concentration during late pregnancy was higher in pregnant women who ate > 150 g fish per day. However, we found no difference in Hg level according to *GSTM1* or *GSTT1* genotype.

Table 4 shows the relationship between Hg level and birth weight according to *GSTM1*/*GSTT1* genotype. Overall, we found an inverse relationship between birth weight and maternal and cord blood Hg levels. The birth weight of newborns whose mothers had the *GSTM1* null type decreased with increasing cord blood Hg level. Birth weight also decreased with increasing maternal Hg level in late pregnancy in newborns whose mothers had the *GSTT1* null type. However, we found no significant inverse relationship between Hg level and birth weight in the *GSTM1* and *GSTT1* present genotypes. In early pregnancy, the birth weight decreased with increasing Hg level but not significantly, and *GSTM1* or *GSTT1* genotype did not affect the relationship. We found similar results that did not reach statistical significance when we carried out multivariate analysis after excluding the preterm births.

Table 5 shows joint effect estimates for Hg level and *GSTM1*/*GSTT1* polymorphisms. Subjects whose Hg levels in late pregnancy exceeded the 90th percentile had significantly lower birth weight compared with subjects with Hg levels less than the 90th percentile. The interaction term for *GSTM1* and Hg level in early pregnancy was significant, whereas the interaction of *GSTM1* and Hg level during late pregnancy was marginally significant. We also found that the interaction between cord blood Hg and *GSTM1* genotype was marginally significant. On the other hand, the interaction between Hg level in early or late pregnancy and *GSTT1* genotype was not significant. In addition, we found no significant interactions between cord blood Hg and *GSTT1* genotype.

Table 6 shows the results of combined analysis considering the *GSTM1* and *GSTT1* genotype simultaneously. In both present types, Hg exposure in the mother and neonates was not associated with birth weight. For either null type, Hg level in early pregnancy was related to a decrease in birth weight but not with Hg level in late pregnancy or in the cord blood. In the case of both null types, Hg levels in maternal blood during late pregnancy and cord blood were inversely associated with birth weight. We found similar results among the subsample of infants excluding preterm births, but statistical significance was not reached.

**Figure 2:**
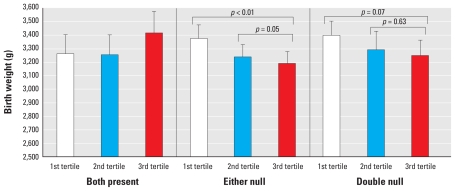
Estimated mean birth weight by maternal Hg tertile and *GSTM1*/*GSTT1* genotypes. Least-square means were estimated in the generalized linear model, adjusting for gestational age, prepregnancy BMI, weight gain during pregnancy, maternal age, education, infant sex, and parity. The average Hg level during pregnancy was divided into tertiles, and genotype was classified by combination of *GSTM1* and *GSTT1* genotypes.

We found significant differences in the least-square means of birth weight according to the combination of *GSTM1* and *GSTT1* polymorphisms and Hg exposure (Figure 2). We found decreased birth weight according to increasing Hg level in women with either a *GSTM1* or a *GSTT1* deletion. Among the women carrying either deletion, we found a significant difference in the least-square means of birth weight by maternal Hg level during pregnancy (i.e., first tertile or second tertile vs. third tertile Hg). In addition, we observed a marginally significant decrease in birth weight in women with a *GSTM1*/*GSTT1* double deletion and high blood Hg level, compared with women with a double deletion and low blood Hg level.

## Discussion

In this study we found that a GST polymorphism may modify the relationship between prenatal exposure to Hg and birth weight. Hg concentration in maternal blood and cord blood was significantly associated with a decreased birth weight. In particular, this tendency was more obvious in women who were *GSTM1*/*GSTT1* double null. Even a small shift in the mean birth weight curve toward the left could cause a public health burden, which would be enhanced among these susceptible groups.

To the best of our knowledge, this is the first study to demonstrate gene–environment interactions in the association between Hg exposure and birth weight.

In this study, the GMs of the total Hg concentrations in the maternal blood during early and late pregnancy were 3.67 μg/L and 3.30 μg/L, respectively. In contrast, the GM of Hg levels in cord blood was 5.53 μg/L, which is higher than that of the maternal blood. The blood total Hg concentration in this study was relatively high compared with concentrations reported in other studies. In a recent study performed in the United States, Lederman et al. (2008) reported a GM total Hg level for maternal and cord blood of 1.6 μg/L and 4.44 μg/L, respectively. Among pregnant women living in Quebec, the GM of blood total Hg level at delivery was 0.48 μg/L (Morrissette et al. 2004). Jedrychowski et al. (2007) reported an average total Hg concentration of 0.83 μg/L in mothers at delivery and 1.09 μg/L in newborns. In contrast, the GM of the blood total Hg concentration in pregnant women in Taiwan and Greenland was 8.6 μg/L and 12.8 μg/L, respectively (Bjerregaard and Hansen 2000; Hsu et al. 2007). In the present study, 13.2% of the maternal blood Hg levels during late pregnancy exceeded 5.8 μg/L, which is the value recommended by the U.S. Environmental Protection Agency to monitor Hg concentrations in blood. Our finding of the higher Hg concentrations in the cord blood than in the maternal blood was consistent with previous studies. This finding can partly be explained by the fact that Hg is transferred to the fetus through the placenta (Kajiwara et al. 1996; Morrissette et al. 2004) and has a high affinity to fetal hemoglobin (Hsu et al. 2007; Iyengar and Rapp 2001), which is consistent with the observation that newborns show a larger hematocrit than do their mothers (Stern and Smith 2003).

The relationship between Hg exposure and pregnancy outcomes is controversial, particularly for low birth weight. Only a few studies have examined these associations, and the published results are inconsistent. Sikorski et al. (1986) assessed the concentration of total Hg in the hair of newborns and found that infants with a scalp hair Hg concentration of > 0.07 mg/kg had a 361-g lower mean birth weight than did infants with lower concentrations. In Greenland, Foldspang and Hansen (1990) reported that higher maternal and offspring MeHg blood concentrations were associated with low mean birth weight. A German study found that exposure to Hg among occupational workers is associated with growth retardation of their newborns (Seidler et al. 1999). These findings are in agreement with the present result that total Hg in maternal and cord blood is associated with decreased birth weight. In addition, in animal studies, neonates from dams exposed to higher levels of metallic Hg (Hg^0^) weigh significantly less than the controls at birth (Morgan et al. 2002), and average weights of the pups at birth were 7–10% lower in groups treated with MeHg during gestation compared with controls (Beyrouty and Chan 2006).

However, some studies do not support the relationship between Hg exposure and birth weight. In a fishing community in Denmark, total Hg concentration in cord blood was not correlated with birth weight (Grandjean et al. 2001). Lederman et al. (2008) found that total Hg in neither cord nor maternal blood was related to newborn size. A recent British study showed that total Hg levels in umbilical cord tissue were not related to birth weight (Daniels et al. 2007). This disparity among studies might be due to differences in genetic predisposition, dietary patterns, and environmental factors (Lederman et al. 2008).

In the present study we investigated the gene–environment interactions between blood Hg and *GST* polymorphisms on birth weight. The gene–environment interaction between Hg level in early pregnancy and *GSTM1* polymorphism was significant. We also found a marginally significant interaction with Hg level during late pregnancy. Because of the half-life of Hg, these results suggest that early pregnancy exposure might be important even though most birth weight gain is attained in the third trimester. Although we performed stratified analysis according to *GSTM1*/*GSTT1* genotype, the decrease in birth weight was significantly associated with cord blood Hg levels in mothers who were *GSTM1* null as well as with maternal blood Hg level during late pregnancy in mothers who were *GSTT1* null. After excluding preterm births (3.4%) from the data, we found similar trends, but statistical significance was not sustained. To date, however, there are no reports of the relationship between Hg levels and birth weight according to GST genotype. Only a few studies observed a relationship between a GST polymorphism and Hg concentration. Gundacker et al. (2007) found that a double-deleted genotype for *GSTT1* and *GSTM1* was associated with a higher hair Hg concentration. In this study, we found no significant difference in the maternal Hg levels in women with either a *GSTM1* or a *GSTT1* polymorphism. Similarly, Custodio et al. (2004) reported that *GSTM1* and *GSTT1* polymorphisms did not affect erythrocyte Hg concentrations.

The mechanism for how a GST polymorphism modifies the relationship between Hg level and birth weight has not been elucidated. Hg has a high affinity for GSH, and binding and dissociation of the GSH–Hg complex are believed to control the movement of Hg as well as its toxic effects in the body (Clarkson 1997). Among the GSH-related enzymes, GSTs may catalyze the intracellular binding reaction of Hg with GSH (Engström et al. 2008; Gundacker et al. 2007). In particular, the roles of the two GST genes, *GSTT1* and *GSTM1*, are important in Hg metabolism because the deletion type of *GSTM1*/*GSTT1* reduces catalytic activity, leading to the slower elimination of Hg (Custodio et al. 2005; Hayes and Strange 2000). In the present study we found that the total Hg level in maternal blood during late pregnancy or in cord blood was associated more significantly with a decreased birth weight in women with the double-deleted genotype than in women with the intact genotype or one of the null genotypes. The double-deletion polymorphism might be more important because this combination impairs the ability for detoxification more and increases the susceptibility to Hg exposure (Gundacker et al. 2007).

Another plausible mechanism is a decrease in birth weight via oxidative stress according to Hg exposure. Hg has been reported to cause oxidative stress, which may lead to lipid peroxidation and the generation of reactive oxygen species (Huang et al. 2008; Pinheiro et al. 2008). Hui et al. (2001) suggested that heavy metals, such as Hg and cadmium, might induce oxidative stress caused by changes in the GSH and/or ATP metabolism. Also, pregnancy is a condition that favors oxidative stress, mainly due to the mitochondria-rich placenta, which may also be enhanced by the presence of metal toxins (Casanueva and Viteriy 2003; Chen and Lin 1998). However, we found no effect of *GSTM1*/*GSTT1* genotype on the relationship between maternal and cord blood Hg. This implies that an effect of *GSTM1*/*GSTT1* on the association between Hg and birth weight might be mediated by genetic modification on the oxidative stress level induced by Hg rather than an effect of *GSTM1*/*GSTT1* on the metabolism of Hg.

The major strengths of this study are the prospective cohort design, the collection of more reliable data from the medical records, and adjustment for important confounders potentially affecting Hg exposure and birth weight. We measured Hg levels in maternal and cord blood, which reflects the changes in recent exposure (Foldspang and Hansen 1990; Grandjean et al. 2001; Lucas et al. 2004). Nevertheless, Hg concentrations assessed in the first and third trimesters could reflect the exposure during early and late pregnancy because the mean half-life of MeHg in blood is approximately 45 days (range, 20–70 days) (Clarkson 1993; Smith and Farris 1996). Information on fish consumption was collected using 24-hr recall, which can result in recall bias and may not reflect dietary intake during the entire period of pregnancy. However, there did not appear to be any differential recall bias, because the dietary survey was carried out before the pregnancy outcome (Xue et al. 2007). In this study, the total Hg level in maternal and cord blood was assessed. Hence, the effect of specific forms of Hg could not be investigated. Although the blood Hg level did not consist entirely of MeHg, it is generally used to represent MeHg exposure because total Hg concentrations in whole blood reflect exposure to organic Hg, predominantly MeHg (Sanzo et al. 2001). Furthermore, cord blood Hg was almost entirely MeHg because the placenta does not present a barrier to MeHg (Kelman et al. 1982).

This study showed that GST gene polymorphisms may play important roles in the relationship between Hg level and birth weight. Although a single defective gene might be associated with decreased birth weight, these results suggest that a combined genotype of *GSTM1* and *GSTT1* should be considered. Therefore, these findings highlight the need for new policy guidelines to reduce the level of Hg exposure in high-risk groups, particularly pregnant women with genetic susceptibility.

## Figures and Tables

**Table 1:** Characteristics of the mothers and infants.

		Birth weight (g)		
Characteristic	*n* (%)	Mean	95% CI	*p*-Value
Maternal characteristics				
Age (years)				
< 30	187 (44.8)	3313.0	3250.6–3375.5	0.22
30 to < 35	178 (42.8)	3273.9	3217.7–3330.0	
≥ 35	52 (12.5)	3200.6	3057.3–3343.8	

Prepregnancy BMI (kg/m^2^)				
< 18.5	55 (13.3)	3170.5	3073.4–3267.5	0.02
18.5 to < 23.0	233 (56.4)	3274.4	3222.6–3326.2	
≥ 23.0	125 (30.3)	3361.2	3277.6–3444.8	

Education (years)				
< 12	135 (33.7)	3275.7	3197.9–3353.4	0.94
≥ 12	265 (66.3)	3279.0	3229.3–3328.7	

Fish consumption (g/day)				
≤ 150	344 (90.0)	3267.3	3221.8–3312.9	0.17
> 150	39 (10.0)	3367.1	3230.1–3504.0	

*GSTM1*				
Present	169 (40.5)	3289.6	3225.2–3353.9	0.77
Null	248 (59.5)	3277.3	3224.5–3330.2	

*GSTT1*				
Present	195 (46.8)	3262.8	3199.4–3326.1	0.37
Null	222 (53.2)	3299.4	3246.6–3352.3	

Infant characteristics				
Sex				
Male	207 (49.6)	3315.8	3253.9–3377.6	0.11
Female	210 (50.4)	3249.3	3196.1–3249.3	

Parity				
0	109 (28.1)	3292.7	3215.6–3369.8	0.99
1	177 (45.6)	3287.9	3218.6–3357.2	
≥ 2	102 (26.3)	3282.8	3211.6–3354.1	

Gestational age (weeks)				
< 37	14 (3.4)	2512.1	2168.3–2855.9	< 0.01
≥ 37	403 (96.6)	3309.0	3270.8–3347.3	

Birth weight (g)				
< 2,500	7 (1.7)	2074.3	1513.4–2635.2	< 0.01
≥ 2,500	410 (98.3)	3302.9	3265.2–3340.7	

Numbers within subgroups vary slightly because of missing values for some variables.

**Table 2:** GMs and percentiles of total Hg concentration (μg/L) in maternal and cord blood.

			Percentile				
Sample	GM	Range	10th	25th	50th	75th	90th
Maternal blood							
Early pregnancy	3.67	0.27–22.6	1.79	2.59	3.85	5.20	6.65
Late pregnancy	3.30	0.12–18.5	1.80	2.45	3.23	4.45	6.27
Cord blood	5.53	0.23–24.1	3.10	4.19	5.54	7.59	9.57

**Table 3:** Distribution of maternal and cord blood Hg concentration (μg/L) by maternal characteristics.

	Maternal blood Hg					
	During early pregnancy		During late pregnancy		Cord blood Hg	
Characteristic	Median	*p*-Value^a^	Median	*p*-Value^a^	Median	*p*-Value^a^
Age (years)						
< 30	3.96	0.85	3.20	0.13	5.62	0.26
30 to < 35	3.84		3.35		5.64	
≥ 35	3.57		3.04		5.06	

BMI at prepregnancy (kg/m^2^)						
< 18.5	3.54	0.66	3.83	0.28	6.03	0.50
18.5 to < 23.0	3.66		3.19		5.48	
≥ 23.0	4.13		3.37		5.74	

Education (years)						
< 12	3.66	0.27	3.09	0.04	5.01	< 0.01
≥ 12	3.89		3.38		5.78	

Fish consumption (g/day)						
≤ 150	3.76	0.32	3.16	0.03	5.54	0.18
> 150	4.27		3.95		5.75	

*GSTM1*						
Present	3.90	0.77	3.30	0.90	5.53	0.88
Null	3.69		3.21		5.58	

*GSTT1*						
Present	3.95	0.70	3.22	0.53	5.54	0.42
Null	3.67		3.26		5.56	

a *p*-Value calculated by the Wilcoxon rank sum test or Kruskal–Wallis rank sum test because of the skewness of the Hg data.

**Table 4:** Regression coefficients and 95% confidence intervals for Hg level associated with birth weight according to *GSTM1*/*GSTT1* genotype.

Exposure period of total Hg (log scale of μg/L)	Genotype			Adjusted^a^			
		Unadjusted		Full sample		Subsample excluding preterm births	
		β	95% CI	β	95% CI	β	95% CI
Early pregnancy	Total	−4.4	−79.5 to 70.8	−68.6	−138.6 to 1.5	−51.4	−122.2 to 19.5
	*GSTM1* present	66.8	−55.6 to 189.2	−37.5	−154.6 to 79.7	−37.8	−155.4 to 79.8
	*GSTM1* null	−48.3	−143.9 to 7.3	−79.5	−168.9 to 9.9	−47.9	−138.8 to 43.0
	*GSTT1* present	9.1	−109.7 to 128.0	−50.7	−158.7 to 57.3	−24.0	−137.8 to 89.9
	*GSTT1* null	−13.7	−110.2 to 82.7	−70.2	−166.5 to 26.0	−63.8	−157.9 to 30.3
Late pregnancy	Total	−27.1	−103.1 to 49.0	−65.5	−135.6 to 4.5	−40.1	−110.9 to 30.0
	*GSTM1* present	39.2	−89.1 to 167.5	−34.3	−153.7 to 85.1	−22.7	−144.6 to 99.3
	*GSTM1* null	−64.4	−159.3 to 30.4	−84.8	−171.4 to 1.7	−57.3	−143.8 to 29.3
	*GSTT1* present	3.3	−124.6 to 131.1	−25.6	−140.4 to 89.2	−5.7	−122.4 to 111.0
	*GSTT1* null	−49.1	−142.1 to 44.0	−99.0	−189.2 to −8.8	−66.8	−156.7 to 23.1
Cord blood	Total	−33.0	−116.1 to 49.9	−86.4	−163.1 to −9.7	−65.2	−143.0 to 12.5
	*GSTM1* Present	54.9	−88.9 to 198.6	−68.3	−206.6 to 70.1	−59.6	−199.5 to 80.3
	*GSTM1* null	−78.5	−180.5 to 23.5	−107.3	−199.7 to −14.8	−74.7	−168.5 to 19.2
	*GSTT1* Present	−39.5	−162.2 to 83.1	−77.7	−187.6 to 32.3	−54.9	−168.2 to 58.4
	*GSTT1* null	−30.5	−144.3 to 83.4	−101.0	−212.3 to 10.4	−76.5	−187.6 to 34.6

a Multivariate regression models included gestational age, prepregnancy BMI, maternal age, mother’s educational level, infant sex, parity, and weight gain during pregnancy as covariates.

**Table 5:** Regression model testing main and interactive effects of blood Hg (μg/L) and *GSTM1* or *GSTT1* genotype on birth weight.

	Early pregnancy		Late pregnancy		Cord blood	
Model	β	*p*-Value	β	*p*-Value	β	*p*-Value
Model 1: *GSTM1* interaction						
Hg level (≥ 90th vs. < 90th)	−114.9	0.18	−197.6	0.01	−215.4	0.007
*GSTM1* (present vs. null type)	−339.3	0.04	−272.8	0.07	−260.4	0.08
Interaction term of GSTM1*Hg level	298.5	0.04	243.8	0.06	231.3	0.07

Model 2: *GSTT1* interaction						
Hg level (≥ 90th vs < 90th)	−73.7	0.40	−80.6	0.34	−64.8	0.43
*GSTT1* (present vs. null type)	−196.8	0.22	137.6	0.39	152.5	0.30
Interaction term of GSTT1*Hg level	158.0	0.26	−154.9	0.27	−166.3	0.20

Adjusted for gestational age, prepregnancy BMI, maternal age, mother’s educational level, infant sex, parity, and weight gain during pregnancy.

**Table 6:** Association of combined *GSTM1* and *GSTT1* genotype and Hg level with birth weight.

Exposure period of total Hg level (log scale of μg/L)	Genotype			Adjusted^a^			
		Unadjusted		Full sample		Subsample excluding preterm births	
		β	95% CI	β	95% CI	β	95% CI
Early pregnancy	Both present^b^	130.7	−49.8 to 311.1	39.6	−144.8 to 224.0	39.6	−144.8 to 224.0
	Either null^c^	−27.3	−141.0 to 86.5	−129.4	−232.8 to −26.1	−99.2	−206.7 to 8.4
	Double null^d^	−27.6	−143.9 to 88.8	−39.9	−155.4 to 75.7	−26.0	−142.6 to 90.5
Late pregnancy	Both present^b^	104.4	−82.1 to 290.9	46.2	−134.7 to 227.1	46.2	−134.7 to 227.1
	Either null^c^	−33.11	−157.1 to 90.9	−74.8	−186.6 to 37.0	−29.9	−143.9 to 84.1
	Double null^d^	−62.8	−169.2 to 43.6	−102.8	−205.4 to −0.2	−89.1	−193.2 to 15.1
Cord blood	Both present^b^	13.3	−193.0 to 219.7	−13.6	−222.5 to 195.4	−13.6	−222.5 to 195.4
	Either null^c^	−17.6	−138.9 to 103.6	−93.9	−203.2 to 15.3	−48.2	−161.5 to 65.2
	Double null^d^	−105.0	−239.1 to 29.1	−138.4	−268.8 to −8.0	−117.9	−251.3 to 15.5

a Multivariate regression model included gestational age, prepregnancy BMI, maternal age, mother’s educational level, infant sex, parity, and weight gain during pregnancy as covariates.

b Both *GSTM1* and *GSTT1* is present.

c Either *GSTM1* or *GSTT1* is null.

d Both *GSTM1* and *GSTT1* are null.
